# From needs assessment to usability testing: evaluating the AinoAid™ chatbot for domestic violence support

**DOI:** 10.1186/s12905-025-04202-3

**Published:** 2025-12-12

**Authors:** Catharina Vogt, Stefanie Giljohann, Natalie Köpsel, Sandra González Cabezas, Jarmo Houtsonen, Ainhoa Izaguirre Choperena, Anna Juusela, Emanuel Tananau Blumenschein, Margarita Vassileva

**Affiliations:** 1 Fachgebiet III.1 Kriminologie und interdisziplinäre Kriminalprävention Department Kriminal- und Rechtswissenschaften Deutsche Hochschule der Polizei, German Police University, Zum Roten Berge 18-24, 48165 Münster, Germany; 2 Department of Clinical Radiology Research Group ‘Cognition and Gender’, Universität Münster, Albert-Schweitzer-Campus 1, 48149 Münster, Germany; 3 Asociación Askabide Liberación, Calle Amparo, 1, Bilbao, 48003 Spain; 4 Poliisiammattikorkeakoulu, (Vaajakatu 2), PO BOX 123, Tampere, FI-33721 Finland; 5 Universidad de Deusto, Mundaiz 50, Donostia - San Sebastián, 20012 Spain; 6 We Encourage Oy Ltd, Putousrinne 1 G, Vantaa, 01600 Finland; 7 VICESSE Research GmbH, Paulanergasse 4/8, Vienna, 1040 Austria; 8 Centre National de la Recherche Scientifique, Pacte/IEP - BP 48, Grenoble cedex 9, 38040 France

**Keywords:** Chatbots, Domestic violence, Intimate partner violence, Artificial intelligence, Technology-based interventions, Prevention, Psycho-education, Support system, Help seeking, Barrier

## Abstract

**Background:**

Survivors of domestic violence (DV) often encounter barriers when accessing professional support services. These barriers arise, for example, from uncertainty in identifying violence, limited knowledge of support options, and psychological barriers such as fear or shame. To address these challenges, the European project IMPROVE developed AinoAid™, a data-secure and multilingual website with an integrated chatbot. Developed in collaboration with psychotherapists, the chatbot provides low-threshold, anonymous, empathetic access to information on domestic violence support services, risk assessment and legal procedures. Ultimately, AinoAid™ seeks to serve as a safe, informative gateway to protection and support.

**Methods:**

Employing a mixed-methods design, Study 1 involved interviews with 80 DV survivors from Austria, Germany, Finland, France and Spain to explore their openness and needs concerning an AI chatbot for 24/7 support. Furthermore, Study 2 was a German evaluation survey of 669 users, assessing perceived safety, usability, utility and interaction quality of AinoAid™.

**Results:**

In Study 1, interviewees predominantly reported limited prior chatbot experience, yet expressed remarkably positive attitudes toward the potential use of a chatbot tailored for DV support. They emphasized the importance of data safety, chatbot accessibility and usability, offering suggestions on chatbot content tone and promotion. The evaluation in Study 2 indicated a positive overall rating of the chatbot by users, with perceived safety emerging as the most highly rated feature. Study participants suggested improving the chatbot’s empathy, engagement, input comprehension, follow-up integration, and memory of previous statements. Additionally, users called for more personalized information, actionable guidance and direct links to relevant support services.

**Conclusions:**

Our evaluation confirms that the AI chatbot AinoAid™ functions as a safe, accessible, and effective gateway to protection and support for DV survivors. Its perceived great strength, its impartiality due to its non-human nature, presents, on the other hand, also a significant challenge in meeting the high expectations for human-like, empathetic, and dynamic conversations in such a sensitive context. However, we anticipate that sustained usage and continuous training will even further enhance the AI ability to fully meet the evolving needs of domestic violence survivors.

**Supplementary Information:**

The online version contains supplementary material available at 10.1186/s12905-025-04202-3.

## Background

Violence against women and girls (VAWG) is defined by the Istanbul Convention as “a manifestation of historically unequal power relations between women and men, which have led to domination over, and discrimination against, women by men and to the prevention of the full advancement of women” [1]. In particular, domestic violence (DV) by current or former intimate partners in any form has the potential to be deeply traumatic for those females who experience these acts. The consequences of DV range from physical injuries [2] and insomnia [3] to depression [4] and even suicide [5]. Approximately 47,000 cases of domestic homicides were counted worldwide in 2020 [6]. In the European Union (EU), 17.7% of surveyed females have experienced physical violence or threats and/or sexual violence by an intimate partner in their lifetime [7]. As a consequence, the estimated economic costs of DV are calculated by the Council of Europe [8] to be 555 € per EU citizen annually.

Nonetheless, the epidemic of DV remains largely hidden, as perpetrators actively conceal their actions, for example, by manipulating survivors of DV into believing that they are solely responsible for and deserving of the violence they endure [9]. Thus, a crucial gateway to support lies in enabling survivors to identify violence and act despite doubting their perceptions, their fears and the enormous taboo that surrounds violence in a formally loving relationship. Accordingly, research shows that survivors’ ways to find protection and support are blocked by individual, situational and structural barriers [10]. *Individual barriers* pertain to survivors’ perceptions, expectations and experiences and influence whether survivors are able to seek help. Self-blame, low self-esteem, shame, the perception of violence as a taboo and a private matter or as a “man’s right” lead survivors to belittle violent incidents and their consequences [11]. Survivors may lack knowledge about support services, which leaves them in a passive position [12]. Moreover, the survivors’ ability to seek support is impaired as they are scared, stressed, and traumatized, which decreases their ability to function [13]. *Situational barriers* pertain to circumstances, current conditions, social relationships, etc., that influence whether survivors are able to obtain support. Perpetrator behaviours such as ‘gaslighting’, social isolation, and intimidation keep violence concealed [14]. Similarly, survivors’ spatial and social isolation [15] and their responsibility for their children [16] bind them to stay in abusive relationships. Furthermore, strong social norms, such as acceptance of violence, partnership idealization or fear of stigmatization [17], hinder survivors from breaking the taboo of their situation. Naturally, the consequences that survivors expect to face when disclosing violence to a trusted person, make the violence public or splitting up are extremely fear-laden [18]. This includes the fear of not being believed [19], of being ignored, of not being taken seriously, of unpleasant questions, in addition to the fear of consequences such as losing social capital, becoming socially isolated [20], or suffering other social repercussions [21]. *Structural barriers* are system-related obstacles that affect the provision and quality of protection or support services day-by-day or in crisis situations [22]. Some survivors carry negative perceptions and experiences of society, support systems and institutions that lead to a low level of trust [23]. Those with a migration history may fear that they lose their residency permit when seeking help [24]. In addition, the ‘Group of Experts on Action against Violence against Women and Domestic Violence’ (GREVIO) [25] regularly criticizes the inadequate availability and distribution of services. Thus, survivors need to be informed about what to expect from which service to assert their rights when faced with a lack of understanding or proactivity [26].

In summary, women affected by DV find themselves in a vulnerable position, which might even be exacerbated by marginalization and intersectionality. Thus, DV survivors need 24/7-available support and valid information as well as empowerment and encouragement to dare to seek and receive support. In many locations, networks of support services exist [27], but survivors need more information and support to find the gateway and overcome the threshold between being victimized and taking concrete action. Owing to their vulnerability, it is important that their autonomy in decision-making is maintained such that they can choose their pace and their direction of help seeking, where they do not have to fear unpleasant questions. This aspect is particularly relevant for individuals who have experienced severe trauma.

The aim of our research and innovation project is to harness artificial intelligence (AI) to provide survivors, as a particularly vulnerable group, with a low-threshold gateway to protection and support. AI has the potential to overcome, mitigate or circumvent the manifold barriers DV survivors face. Nonetheless, the current literature has critically examined, whether AI can provide a positive solution, and in what ways. Studies indicate that DV survivors express concerns about the indispensability of human support, in addition to issues of safety and security concerns. Empathic connections and trust-building are more difficult to establish due to the absence of social cues, and technological challenges may also arise. These include issues of connectivity and privacy, simultaneously creating the need for access to technology and the internet [28, 29]. While some survivors do not have access to technology, other minoritized or marginalized survivors (e.g., neurodiverse, older, deaf, or struggling with literacy) may face barriers to accessing technology without further help [30]. In parallel, it also highlights its potential for anonymity, a non-judgemental approach, immediate, accessible responses, overcoming language barriers, the provision of additional avenues to safety, and the opportunity to establish a central recording system for criminal processes, and concludes that it may be “better than nothing” [29–31]. Although some persons might be reluctant to use text-based services, most individuals do not differentiate in their disclosure preferences between an AI and a human counterpart [32]. After their introduction, some text-based crisis hotlines are used as often as telephone hotlines [31, 33]. When provided in a timely manner and in ways that properly identify users’ needs and options, technology can be a valuable support and virtual front door to services [29, 34]. By offering information on resources and support, and by contributing to survivors’ understanding of their situation and to their improved safety, it strengthens access to help. This pertains especially to young persons and those living in remote areas, as well as to situations where direct human contact might not be feasible or accessible (e.g., due to transportation problems, child-care responsibilities, lack of time or pandemic conditions). In this way, more survivor groups can access the support system, for instance, individuals who want to avoid the stigma of seeking help or who do not wish to be seen doing so [28, 29, 34–36].

Accordingly, digital services need to be effective in rapport building and therefore must mimic unconditional positive regard, active listening, asking questions, paraphrasing, validation, and evaluative language specifically tailored to the user input [28]. Moreover, digital service in the field of DV need to accurately recognize users’ expressed sentiments or emotions – particularly in those with depressive symptoms – and react appropriately, while also conducting risk detection [37]. In parallel, service providers also need to support these tools to encourage their use in practice [38],, e.g., by ensuring that clients use high-quality, trusted, and secure digital tools if they discontinue using the providers’ services. In this regard, the psychological risks of AI, arising from misleading pseudo-intimacy, have also been widely discussed, as they pose a particular threat to users who experience isolation or psychological distress [39]. The listed risks are manifold, ranging from commodified intimacy, emotional over-attachment, emotional dependence, emotional delegation, the replacement of relational labor by algorithmic simulation, manipulative engagement, and reinforcement of social isolation, to risks related to data extraction, consent and boundary violations, ethical roleplay violations, deceptive outputs, and potential misuse. Further risks include biased training data or misinterpretations, lack of maintenance and development of the technology (e.g., outdated content or broken links), and ethical risks arising from vulnerable users or unregulated design, which can lead to unintended consequences [30, 37, 39–41].

To manage these risks, digital services must be critically and regularly evaluated for their potential to undermine users’ emotional capacity for action and for their safeguards that preserve transparency and accountability, ensuring that users are not exposed to further risks (e.g., perpetrators’ control of survivors’ devices for abusive reasons) [30, 39]. Nonetheless, research shows that users of human-like chatbots indicate that, despite these risks and potential harms, these digital “relationships” are beneficial to their well-being. The more consciously human-like the chatbot is perceived, the more positive opinions and pronounced social health benefits are reported [42].

Responding to recent research appeals [43, 44] and a call by the EU [45] to develop an AI chatbot for DV survivors as an intervention to bridge the gaps between victimization and active help-seeking, we developed the *AinoAid™ chatbot* [46] as part of the larger EU project ‘Improving Access to Services for Victims of Domestic Violence by Accelerating Change in Frontline Responder Organisations’ (IMPROVE) [47], which we introduce in the following section. Our primary research objectives are as follows:

1. The needs, expectations and concerns of potential chatbot users regarding chatbot usability are explored via an interview study with DV survivors (Study 1), and the aforementioned barriers that DV survivors face are addressed by introducing the AinoAid^™^ chatbot as a gateway between survivors and the help system.
2. The chatbot users’ evaluation of the chatbot’s usability with previously identified needs is investigated (Study 2).

## The AinoAid™ chatbot

AinoAid™ [46] is an AI-based service designed to provide psycho-social support and guidance for survivors of domestic violence (DV), acting as a gateway to protection and support, as well as assisting frontline professionals in offering effective support and guidance. Moreover, AinoAid™ is going to be enlarged to further VAWG content within the next 2 years in the framework of the REACH project. This in particular pertains to content on sexual violence, forced marriage, and human trafficking.

The core premise behind the design of AinoAid™ was to create an accessible, reliable, and trustworthy resource where users can find, access, and utilize information on DV [44]. AinoAid™ [46] is compatible with smartphones, tablets, and computers and does not require any downloads. The service consists of two main components: (1) an AI-based chat designed to provide support and guidance and (2) a knowledge base that offers valuable information. AI-based chats aim to provide psycho-social support by mimicking human conversation, and were co-created and curated with input from DV survivors, therapists, and psychologists to reflect interactions typically found in therapeutic settings [43], with strict adherence to safety and ethical principles. The content of the knowledge base, created by DV experts from the IMPROVE consortium, can be accessed either through the chat interface or independently without engaging in a conversation. It includes frequently asked questions (FAQs), safety guidance, detailed information on various forms of violence and on how to recognize these, instructions on seeking help, information on legal and criminal processes, on accessing shelters and support services. The platform also offers checklists, a safety plan template, a self-risk assessment questionnaire and contact information for emergency services and other relevant organizations, such as mental health support.

AinoAid™ has been launched in Austria, Finland, La Réunion, Germany, and Spain. Accordingly, the content has been tailored to the specific national contexts and DV-related legislation. When accessing AinoAid™, users are first prompted to select their country and then the language they wish the platform to refer to [46]. When a user queries the chatbot, it accesses the vector database linked to the Sanity CMS [48], fetching the most relevant content (i.e., operating on a Retrieval Augmented Generation/RAG approach). The system prompt then adds instructions regarding tone and style (e.g., empathetic, supportive), and the language model combines the user’s message, the chat history, and the retrieved content to generate a response. If the risk-classification or intent-detection models flag the need, a contact card (with helplines and local services) is added automatically. The prompting instructions ensure that the model’s tone remains empathetic, professional, and supportive. In addition, established processes for continuous monitoring and feedback collection during piloting and beyond enable concerns about tone or user-friendliness to be identified early and addressed promptly. This ongoing monitoring helps the chatbot to respond to users’ needs in a manner that is both effective and sensitive.

### AinoAid™ chatbot technology: High-level architecture design

The design of the AinoAid™ architecture was developed through multiple iterations. Initially, the starting point was a chatbot. However, as the needs of DV survivors became more apparent, the architecture evolved from a simple rule-based chatbot to a more comprehensive system that includes a knowledge base, which enables users to access the information also without chatbot interaction.

The high-level solution design is modular, encompassing content production and publishing, as well as the DV survivors’ user service (front-end, i.e., the AinoAid™ website). This approach focuses on user journeys and provides various entry points for information search and chat functionalities. Modularity is essential for avoiding vendor lock-in, ensuring cost-efficiency, and enabling future upgrades as technology evolves. Additionally, the ability to enhance content and leverage user data for future service development is critical to the scalability of the service.

**Fig. 1:**
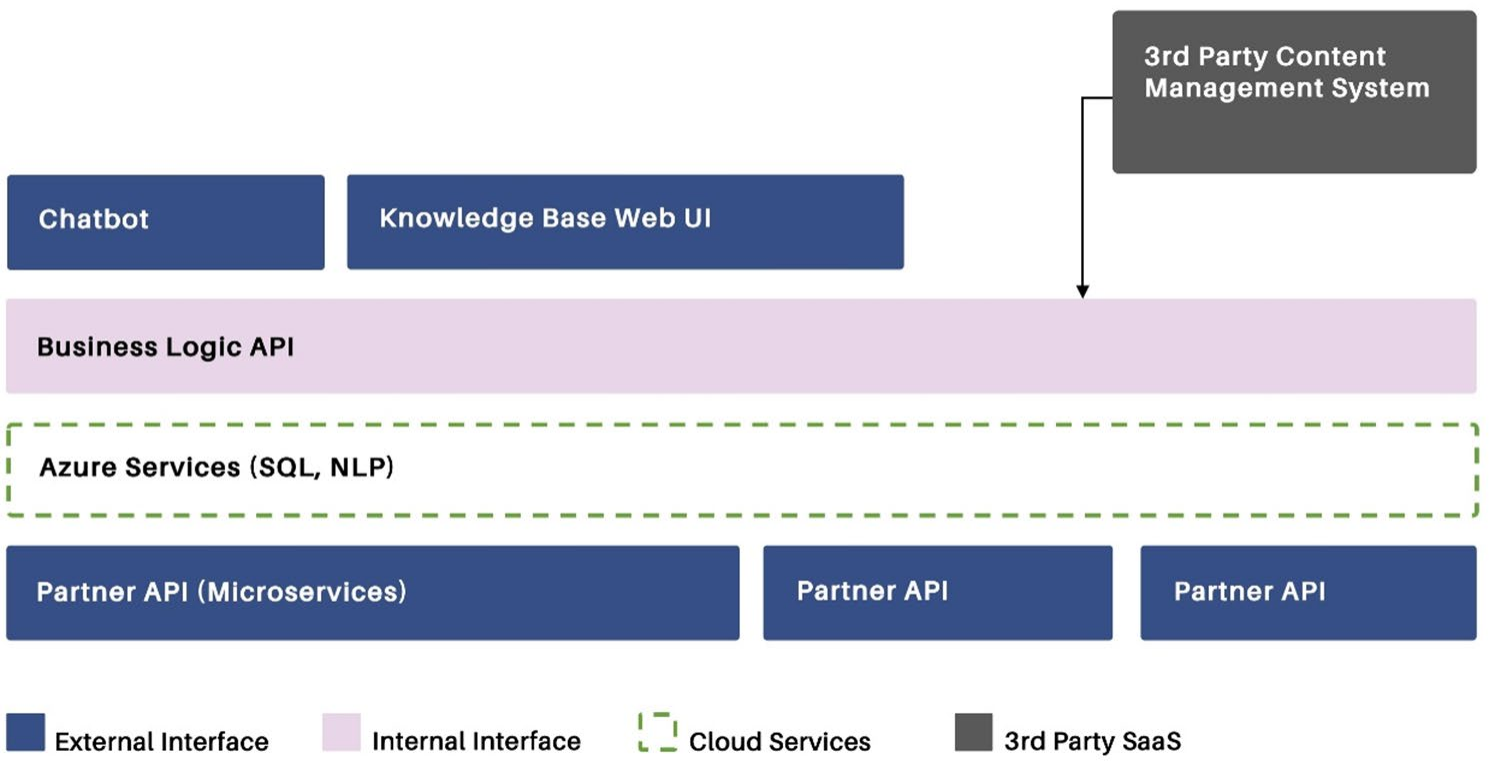
High-level architecture of AinoAid™ (© WeEncourage Oy Ltd., permission to be published in this paper)

In addition to the high-level architecture design (Fig. 1), detailed functional requirements for AinoAid™ were prioritized, finalized, and reviewed by the IMPROVE consortium members for approval before moving to the implementation phase. The high-level architecture design provided the foundation for selecting the most cost-effective methods to build the chatbot prototype while ensuring continuity of development beyond the IMPROVE project [47]. The main services, knowledge base, website, and chatbot are built using Azure [49] services, with the chatbot based on Azure Bot Framework technology. While it is specifically trained in English, Finnish, French, German (Austria and Germany), and Spanish, it is multilingual because of the integration of the Azure Translator. The knowledge base, i.e., the AinoAid™ website [46], is built on a branded custom application utilizing the content management system (CMS) Sanity [48].

### AinoAid™ chatbot content management

**Fig. 2:**
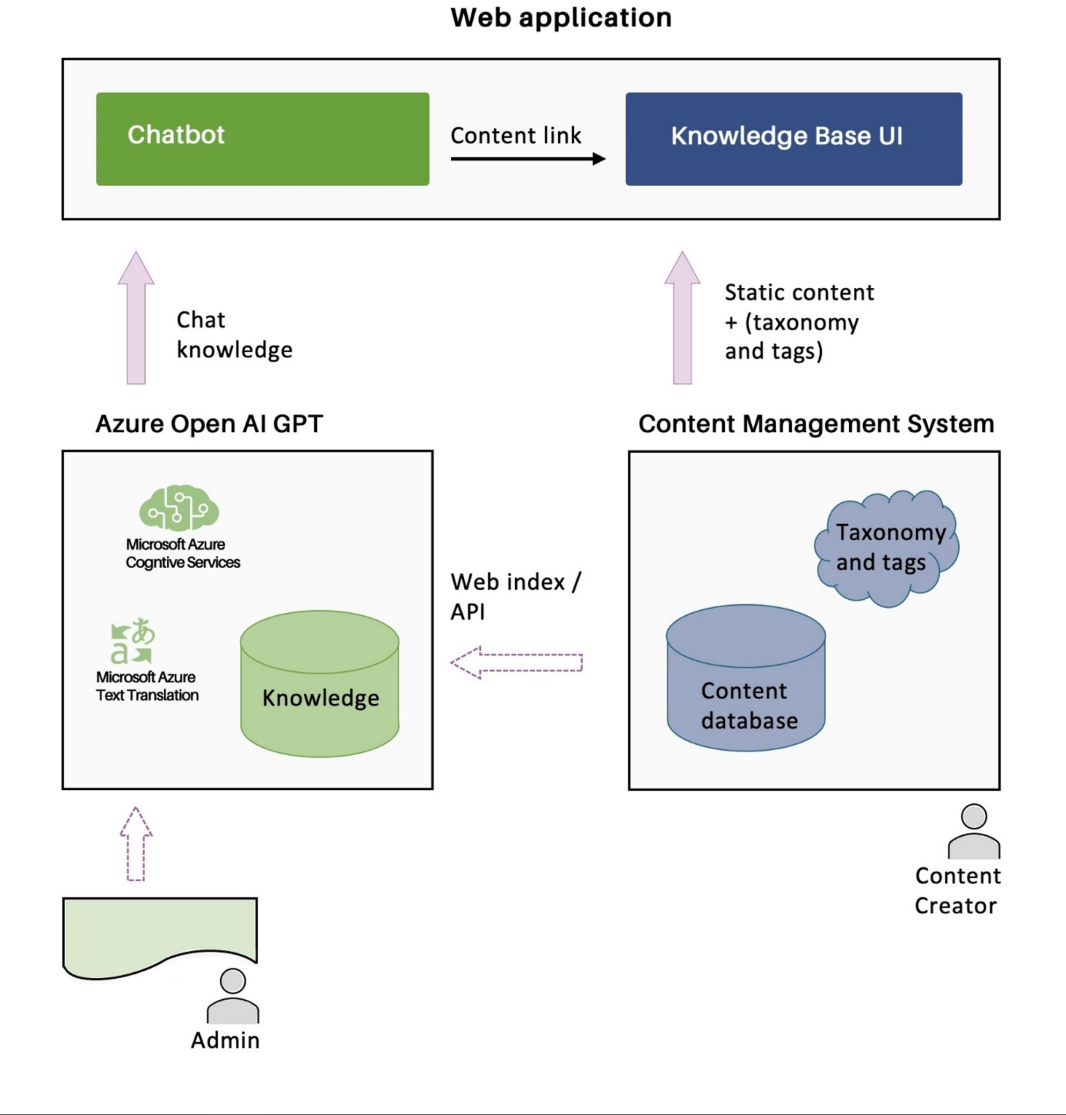
Basic information flows in the AinoAid™ service (© WeEncourage Oy Ltd., permission to be published in this paper)

The simplified diagram (Fig. 2) illustrates the basic information flows in the service. The chatbot operates on the basis of its available content, which is either manually fed via Azure or provided through the Sanity CMS application programming interface (API) [48], the primary source of the content. Sanity CMS is used to create and publish content to the knowledge base website. The front-end provides the end-user interface, which is compatible with any device. The front layer is not restricted by any specific technology or product but is built via modern frameworks. As a result, the chatbot retrieves its information from the knowledge base only, ensuring the delivery of reliable, unbiased content.

### AinoAid™ chatbot safety: data privacy and security

The AinoAid™ development team closely collaborated with Microsoft’s (MS) engineering team on technology and designed decisions to ensure compliance with MS’s AI ethics guidelines. In the service, user data privacy is prioritized through various technology components in several ways:

1. Technology such as the Azure environment for storing user conversation data was selected on the basis of data privacy specifications and Azure’s compliance with a wide range of external privacy standards, laws, and regulations, including the General Data Protection Regulation (GDPR), ISO/IEC 27,701, ISO/IEC 27,018, and EU Standard Contractual Clauses, with data stored within the EU.
2. Personal data are neither stored in databases nor processed, meaning that GDPR enforcement is not applicable. No internet protocol (IP) addresses, personal names, identification numbers, email addresses, or street addresses are stored or processed. In cases where survivors inadvertently include such personal data in their chat interactions, Azure’s Personal Identifiable Information (PII) detection tool is used to remove it [50]. Consequently, Azure PII ensures the removal of any PII from the chat history.
3. All the data are securely stored and accessible only through specific Azure access rights, ensuring robust data protection.
4. Confidentiality and availability: Data from the service are accessible only to authorized parties, and access is granted solely for specific purposes and when necessary.
5. The knowledge base includes a privacy statement, which can be found in the privacy policy section of the AinoAid™ website.
6. Data Protection Impact Assessment (DPIA) requirements were reviewed, and the decision was made not to conduct a DPIA.

In addition, the potential risk of AinoAid™ being misused by violent partners against their victims is mitigated in two ways: On the one hand, the knowledge base information and chatbot response are prepared in a way that avoids providing instructions that could increase the risk to survivors. Instead, the chatbot provides assistance in recognizing violent behavior and guides individuals using violence toward responsible support services, with the aim that any engagement with the tool contributes to prevention and rehabilitation, rather than causing harm. A clear stand against the use of violence is taken. On the other hand, users are made aware and guided by the lead section under “FAQ and Safety” to safeguard their privacy, to use a secure device, to be cautious with personal information, to surf in incognito mode and to clear their browsing history to prevent them from being found out by a perpetrator.

### AinoAid’s capabilities in the fight against DV

The design of AinoAid™ [46] is grounded in the expectations and experiences of DV survivors as well as professional DV expertise and is further developed through continuous updates from IMPROVE consortium members and their networks to ensure the tool remains relevant, responsive, and secure. Thus, the AinoAid™ chatbot [46] represents a cutting-edge technological tool designed to enhance support services, utilize data-driven insights, and facilitate technology-driven interventions, as called for by research [43, 44]. The incorporation of technology into support and protection strategies for women and girls affected by DV presents a significant opportunity to expand the reach and effectiveness of these interventions [51]. When deployed ethically and responsibly, these technological innovations can strengthen support systems, offering safer, more empowering paths for women to rebuild their lives. In this context, the AinoAid™ [46] chatbot has significant potential in terms of anonymity, accessibility, availability, design, and awareness-raising, all of which will be discussed further below.

#### Anonymity and online safety

While there is broad support for using AI and chatbots as initial resources for survivors of DV [52], the chatbot’s anonymity is crucial for its acceptance [53]. Therefore, the chatbot operates anonymously and does not require user registration, which enhances both accessibility and safety. Additionally, users are advised at the start of the chat not to disclose any personally identifiable information (PII) about themselves or others. To support online safety, users are provided with guidelines for safe browsing. A quick exit button, leading to the Google search engine [54], is always visible during both the chat and the navigation of the knowledge base. This comprehensive approach encourages survivors to seek support without fear of being identified or exposed [18, 46].

#### Accessibility and availability

The advantages of chatbots, particularly compared with most conventional help services, such as their 24/7 availability and instant responses, highlight their potential to provide immediate support. For ease of access, AinoAid™ is designed as a standard internet homepage that includes a chat function, meaning that users do not need to register, download anything, or possess advanced digital skills to use it [46]. Survivors only require a computer or mobile phone with internet access. In addition to offering information on necessary support services and contact details, AinoAid™ also facilitates the referral of survivors to face-to-face services. These features make it an especially valuable resource for women and girls, who may need assistance at any time, particularly during crises [22]. As a result, its accessibility can empower individuals to take proactive steps toward addressing their needs and challenges.

#### Inviting design

Persons who experience or are exposed to DV are always at risk of being retraumatized [13]. For this reason, AinoAid™ is designed to be trauma sensitive, featuring a neutral, user-friendly layout that conveys a fear-free, safe, friendly, and welcoming online space. Equal emphasis was placed on language and content during the development of the platform to create a judgment-free environment [53]. Responses are formulated in an empathetic and polite manner, ensuring that users have a positive conversational experience while addressing different aspects of violence and offering support.

In terms of content, the chatbot provides detailed and comprehensive responses that effectively cover various aspects of the topics discussed. AinoAid™ also offers clear, concise, and practical guidance, such as instructions on how to disable location services on phones, ensuring that even stressed, weary, confused, and unfocused survivors can follow the steps [46]. When requested, the chatbot provides extensive information, such as detailed explanations of survivors’ emotions or the impact of violence, and offers checklists and tips to deliver helpful and valuable guidance. In summary, the chatbot’s ability to create an emotionally safe and constructive space for individuals seeking guidance can encourage them to reach out for help, especially in situations where they might otherwise hesitate to do so.

#### Awareness Raising

The chatbot’s ability to raise awareness, facilitate self-identification, and provide educational resources on lesser-known forms of violence, such as psychological and vicarious violence, represents a critical opportunity for both prevention and education. By highlighting these aspects of DV [44], chatbots help survivors recognize the full scope of abuse they may be experiencing or witnessing. Such efforts are essential for shifting cultural narratives, challenging harmful norms, and empowering women to take preventive action before violence escalates. Furthermore, promoting self-care and offering contact details of support services, along with information on how they operate, are intended to foster a positive user experience. Therefore, while the AinoAid™ chatbot [46] holds significant potential as an initial point of contact for DV survivors, to facilitate the reporting of violence, and to help overcome historical barriers by empowering survivors. Its ultimate effectiveness will depend on how well it aligns with the needs of DV survivors and how they assess the service and the support it provides.

## Study overview

To guide the dynamic and iterative development process of AinoAid™ [46], an interview study with DV survivors (Study 1) was conducted in parallel with the initial development phases of the chatbot. The goal was to assess the needs, expectations, and concerns of potential users. The findings from this study were then used to refine AinoAid™’s features, ensuring that they better aligned with the identified needs and expectations of survivors. In a subsequent follow-up study (Study 2), public opinion on the chatbot was surveyed to evaluate how users rated the key features of AinoAid™ that were highlighted as important by participants in Study 1.

### Methods Study 1

In Study 1, we explored the needs, expectations, and concerns of potential chatbot users regarding chatbot usability by an interview study with DV survivors. The recruitment process primarily involved victim support associations. In line with the study’s approach to researching vulnerable groups of DV survivors, the interviewees represented a wide range of marginalized populations. The interviews were conducted in Austria, Finland, France (La Réunion), Germany, and Spain. Table 1 provides an overview of the sample characteristics of the 80 female interviewees.

Data collection was based on semi-structured interviews designed to capture (a) interviewees’ opinions on chatbots in general, (b) whether they would personally use a DV-specific chatbot in case of need, (c) what concerns they would have regarding its usage, (d) which opportunities they see in the idea of such a chatbot, (e) which information the chatbot should provide, and (f) whether they would prefer to write or speak with the chatbot and which voice it should have. The interviews were conducted by trained researchers in neutral settings or by telephone, depending on the interviewees’ preferences. Prior to participation, the interviewees were informed about the research project as well as their rights in terms of voluntariness, anonymity, data processing and data protection. All the participants agreed with the procedure and signed an informed consent form. The interview guide has been uploaded under the supplementary materials section.

To follow ethical research practice and confidentiality, the identity of the interviewees was protected in accordance with the General Data Protection Regulation. All interviews were audio-recorded, anonymized, and summarized (partial transcription) for analysis.

**Table 1:** Sample characteristics (Study 1)

	Austria	Finland	France	Germany	Spain	Sum
Geographic location						
Urban	3	16	6	6	12	*43*
Rural	3	5	4	10	15	*37*
*Special categories of survivors*						
Survivors with mental health problems	-	15	1	5	-	*21*
Survivors with disabilities	-	-	-	3	-	*3*
Survivors with substance abuse problems	-	1	-	-	-	*1*
Elderly survivors	-	-	2	-	2	*4*
Mothers	6	3	5	10	27	*51*
LGBTQI* survivors	-	1	1	1	-	*3*
Migrant survivors	3	3	2	11	4	*23*
Refugees	-	-	-	1	-	*1*
Closed religious community	-	3	-	-	-	*3*
*Total number of interviewees*	*6*	*21*	*10*	*16*	*27*	*80*

We utilized thematic analysis [55] to explore key categories and features to be considered in the development of a DV-specific chatbot, particularly AinoAid™, as a gateway into the support system for survivors of violence. Thematic analysis can focus on the manifest content of conversations and does not require full or verbatim transcriptions [56]. Therefore, only passages relevant to the identified themes were transcribed. This approach involved listening to the audio recordings and selectively transcribing meaningful segments, accompanied by contextual annotations, including the interview identification number/code, timestamp, thematic coding, and contextual details. In a first step, all authors structured the relevant content along three guiding questions in order to present all possible views in a comprehensive manner. These questions addressed (1) interviewees’ attitudes toward an AI chatbot supporting DV victims, (2) their experiences with chatbots, and (3) their expectations and wishes regarding such a chatbot. The authors then analyzed the material, identified sub-themes, and reorganized the interview content into coherent thematic groups based on these sub-themes. This strategy enabled us to systematically evaluate the relevance of each topic. The national teams then translated their coded material from the original interview languages into English for the joint analysis. Finally, the third author condensed and structured the topics into multiple interrelated categories, which were reviewed and validated by the first author in order to clarify their interconnections, particularly regarding (1) experiences and attitudes toward DV-specific chatbot usage, (2) accessibility, (3) need for security and trust, (4) chatbot content, (5) chatbot empathy and encouragement, (6) chatbot design and functionality, and (7) chatbot promotion.

### Results of Study 1

The following sections present survivors’ needs, expectations, and concerns, as reflected in the seven thematic categories of our interview analyses.

#### Experiences and attitudes toward DV-oriented chatbot usage

The majority of the interviewees reported using the internet on a daily basis to seek information across a variety of topics. However, very few of them had knowledge of or experience with chatbot services. For example, a young mother was aware of the French chatbot “Elle Caetera” [57] for young females exposed to sexist or sexual violence. Among the few participants with prior chatbot experience, the interaction was commonly described negatively as automated and lacking personalization. While still expressing a preference for human interaction, most of the interviewees showed a positive, possibilistic attitude toward a DV support chatbot. The interviewees emphasized the high potential and usefulness of such a chatbot, especially in the initial phase of support-seeking, when the need of general information regarding DV and related services is high. As a low-threshold gateway, the chatbot was perceived as a potentially more approachable alternative to human interaction, particularly given the sensitive nature of the topic. Expectations were that such a chatbot should enable survivors to understand what kind of help to seek and where to find it. Beyond the initial contact, it was also considered potentially helpful during later stages of the support-seeking process, for instance, during recovery, by offering encouragement and guidance, such as supporting survivors in their decision not to return to the perpetrator.

#### Chatbot accessibility from a technical and psychological perspective

The interviewees emphasized the chatbot’s 24/7 availability as a major advantage for support-seeking, in addition to its immediate responsiveness and location-independent accessibility. These features were perceived as particularly beneficial by survivors residing in remote areas with limited proximity to support services. Furthermore, the possibility of accessing chatbots in multiple languages without third-party assistance was highlighted as a strength, especially for individuals with language barriers or limited social support. However, participants also pointed out that digital accessibility remains constrained when survivors have insufficient or no access to the internet.

According to the interviewees, the psychologically reassuring aspect of a chatbot lies in its inability to judge, which was perceived as reducing survivors’ fear of rejection and encouraging them to ask questions they might otherwise consider too trivial or inappropriate to pose to professionals. *“A robotic attitude is good*,* because otherwise the victim will have more emotional burden if she feels that she is burdening the listener. The victim senses easily if the listener gets frustrated or starts to blame the perpetrator. In that case the victim may feel a need to defend the other. Victims may sense*,* if they are being blamed for violence. Therefore*,* a facts-as-facts approach would be good.” (I15)*.

In addition, several interviewees considered a chatbot particularly appealing for younger individuals, who they perceived as being more comfortable with written communication and often more reluctant to make phone calls, whereas one interviewee suggested that: “Children and young persons should have their own versions of the chatbot to get more information on domestic violence and how to seek help” (I9).

#### Need for security and trust in the chatbot

The interviewees expressed concerns about uncertainties regarding who they would be communicating with, who might access or monitor their messages, and how their data would be secured. However, very few of them indicated that such concerns would actually prevent them from using a support chatbot. Instead, the necessity of ensuring anonymity was strongly emphasized across all interviews. Anonymity was perceived as key for lowering the threshold to engagement - both for security reasons and to avoid feelings of shame.

Technically, the interviewees stressed that the chatbot must operate in a highly secure and discreet manner, leaving no digital traces. It was suggested that access could be facilitated in public spaces such as bars, settings perceived as safer than one’s own home. This would reduce the risk of discovery by abusive partners or, in cases of insecure migration status, by law enforcement. Similar concerns were expressed for individuals struggling with substance abuse, who may fear disclosing their situation due to potential consequences. According to the interviewees, the chatbot’s infrastructure, functionality, and content must guarantee that users remain completely anonymous and untraceable at all times.

The interviewees also raised the potential risk of misuse, either by others or against survivors, as well as the danger of chatbots generating inaccurate or misleading information. Such “hallucinations” were seen as highly problematic, potentially leading to disappointment or even harm. Hence, the chatbot was expected to deliver only reliable and verified information.

Furthermore, the interviewees underlined the importance of ensuring that chatbot interactions do not re-traumatize users or convey any form of victim-blaming. Interestingly, one interviewee considered this goal more realistically achievable via chatbots than in conversations with social workers, making her prefer the former. Nevertheless, several participants voiced concerns that a chatbot might lack social sensitivity and emotional resonance, potentially making it feel “cold” or detached. Moreover, they cautioned that the AI might fail to recognize or appropriately respond to acute danger situations.

#### Chatbot content

The interviewees shared a wide range of ideas regarding the kind of recent, evidence-based content that should be provided by a support chatbot. They emphasized the importance of information that helps identify violence in various forms, along with guidance on risk factors, risk assessment, and even more specific content such as how to monitor digital abuse. Additionally, the interviewees called for instructions on ensuring safety and information about immediate help options. There was also a clear demand for resources aimed at third-party intervention, such as advice for friends of those experiencing violence, as well as legal information, explanations of which professionals are typically involved in DV cases, and tips on how to access therapy. It was considered important to make the pathways through the support network more transparent: *“Maybe it would be good to include a section on available support and how long each step of the process takes. Many times*,* you don’t know what’s going to happen next.” (A1).* Some participants also warned that not all professionals treat survivors appropriately, highlighting the need for guidance on how to navigate such situations.

In addition to these expectations and suggestions, the interviewees expressed strong interest in diverse, knowledge-enhancing resources. These included, for example, instructions for developing safety plans, self-assessments to reflect on one’s situation and level of risk, and personalized risk evaluations. They also suggested the inclusion of curated book recommendations and audio-visual materials, such as videos, TV series, or films, that could help increase awareness of one’s circumstances and experiences of violence.

Crucially, survivors noted that the chatbot should not only serve as a source of information but also be able to assess a woman’s situation, identify signs of abuse and associated risks, and provide personalized guidance accordingly. The interaction should be responsive to the specific context of the survivor, taking into account, for instance, whether she is still in a relationship, going through a breakup or a divorce. As such, the chatbot must be equipped to respond meaningfully to complex case descriptions and provide adequate clarification when doubts arise. Survivors also expected to be understood on an emotional level and that the chatbot responded to this appropriately and informatively: *“A chatbot would evaluate a person’s emotional state and give tips*,* help her notice how her body reacts and explain how it is a normal reaction in certain situations. By doing so*,* the chatbot would validate a victim’s emotional state and nervous system reactions.” (I18)*.

#### Chatbot empathy and conversational design

The interviewees emphasized that the chatbot’s AI should be tailored toward personalization and empathy, conveying an understanding of survivors’ emotional states so that they feel heard, believe, and receive responses that reflect their individual situations. The chatbot’s communication should offer emotional support by using encouraging and action-oriented language, for example, by posing direct questions or reminding survivors after a break-up of the reasons they ended their relationships. One interviewee illustrated these expectations on the basis of her own experience: *“You have to… not push. Point out*,* but don’t push! That’s exactly the problem. I wasn’t forced. I was just… presented with a lot of possibilities”* (V4).

In line with this, the chatbot should initiate conversations with open-ended questions, deliberately avoiding the explicit term *violence*. Instead, the survivor’s situation should be gradually explored through carefully phrased follow-up questions that sustain user engagement. Additionally, one interviewee proposed offering short testimonies from other female survivors for each type of violence addressed to reassure chatbot users that they are not alone, an approach that may offer emotional relief or encourage them to seek support.

#### Chatbot interface design and functionality

The interviewees described a wide range of characteristics they considered essential for the chatbot’s interface: These features aligned with an overarching vision of straightforward yet inviting functionality. The interface should be clear, concise, well-structured, visually pleasing, and avoid excessive use of color and unnecessary technical elements, such as an overload of buttons or screens. Rather than feeling overwhelmed, survivors should feel “welcomed” by the interface.

Opinions have diverged regarding whether communication with the chatbot should occur primarily via voice or text. While some survivors may lack the strength to type during a crisis, interviewees noted in favor of writing that a written dialog may be perceived as more discreet than speaking by the users. In the case of the implementation of voice communication, the interviewees recommended providing both male and female voice options or allowing users to choose, emphasizing that the voice should be calm and compassionate. Additionally, one interviewee suggested an alternative communication format particularly helpful for traumatized individuals: selecting from predefined responses to progress through the conversation by clicking.

With respect to the chatbot’s functionalities, the interviewees proposed numerous suggestions. These included the possibility of direct communication via video if desired, geo-localization to enable mapping of nearby support services, and automated referral pathways, such as direct links to service booking systems. Some participants suggested memory functions to save conversations for later sharing with professionals, thereby avoiding repeated explanations. Other interviewees drew attention to the value of documentation tools, including the option to upload photos or videos (e.g., of injuries) and send them to third parties such as healthcare providers. Furthermore, some interviewees proposed that third parties, such as professionals or institutions, could receive or transmit relevant chatbot content to survivors.

This wide array of suggestions reflected a broader desire for the chatbot to serve as an effective gateway into the support system - one that not only informs but also facilitates direct access to help. Many interviewees underscored the importance of practical support and referral functions, as articulated by one survivor: *“It would be good if the chatbot could forward the client to a real person in chat but also the chatbot could forward the conversation to a professional (with victim’s consent)*,* who would get information about the client in advance*,* before starting a conversation with the victim”* (I17).

Particularly in high-risk situations, chatbots should guide survivors directly toward relevant services and aid providers in their region. Several participants highlighted the need for an emergency function, such as a dedicated button, that enables immediate contact with emergency services such as the police, hospitals, or shelters.

#### Chatbot promotion

Once developed and implemented, the interviewees strongly expressed the wish that the chatbot would be widely disseminated, particularly in spaces frequently accessed by women. For online dissemination, suggestions included embedding the chatbot on the websites of trusted local domestic violence (DV) service providers, promoting it via social media channels, or integrating it into a commonly used platform such as the GPT chat. In addition to the digital realm, the interviewees recommended a broad public outreach strategy. This could involve advertisements in newspapers and visible placement in public institutions, such as kindergartens, schools, workplaces, healthcare facilities, and even large events such as concerts.

### Methods Study 2

Participants were recruited through a German online panel provided by Dynata [58], which offered credits for participation that could be exchanged for rewards within the panel system. The sample included 669 participants. Age was categorized into six groups. Twenty-six percent of the respondents belonged to age group 1 (18–30 years), 18% to age group 2 (31–40 years), 18% to age group 3 (41–50 years), 19% to age group 4 (51–60 years), 15% to age group 5 (61–70 years), and 4% to age group 6 (71 years or older). A total of 43.5% (*N* = 291) of the respondents reported having personal experience with domestic violence. Of these, 17.2% (*N* = 115) had been personally affected by DV, while 22.9% (*N* = 153) reported having relatives or acquaintances who had experienced DV. Eleven percent (*N* = 76) of participants had encountered DV in a professional context. Thirty-five percent (*N* = 236) reported having previously engaged with the topic of DV in depth, primarily through personal internet research, media consumption (e.g., social media, TV, movies, news, brochures, exhibitions, podcasts), personal or vicarious experience (as a survivor, acquaintance, or colleague), or professional training (e.g., in school, as nurses, or through volunteer work). Five percent of participants (*N* = 35) had used a chatbot other than AinoAid™ to address DV, with the most commonly mentioned being ChatGPT [59], followed by Meta AI [60], Gemini [61], Amy Rose [62], Samsung AI [63], and SOS APP [64].

In accordance with established usability measures [65, 66], we assessed various aspects of chatbot usability, including helpfulness, informativeness, comprehensiveness, and safety. Additionally, we evaluated chatbot language, focusing on tone of voice (simplicity, friendliness, warmth, empathy, and formality), as well as website usability, encompassing design, navigation, and safety. The survey^1^ was developed for this study and has not previously been published elsewhere.

To assess participants’ perceptions of chatbot usability, we asked, “How helpful did you find the interaction with the chatbot?” and “How informative did you find the chatbot?” using 5-point Likert scales, where 1 indicated “very helpful” or “very informative,” and 5 indicated “not helpful at all” or “not informative at all.” We also gauged participants’ sense of safety while using the chatbot by asking, “Did you feel it was safe to use the chatbot?” on a bipolar scale (1 = yes, 2 = no).

Regarding chatbot language, participants were asked, “How did the chatbot’s language style appear to you?” using a 5-point semantic differential scale with the following opposite poles: ‘simple’ versus ‘complicated’, ‘friendly’ versus ‘unfriendly’, ‘warm’ versus ‘cold’, ‘empathetic’ versus ‘distanced’, and ‘pleasantly formal’ versus ‘too formal.’ The positive pole was rated as 1, and the negative pole was rated as 5. Additionally, we asked participants, “How comprehensible did you find the chatbot’s answers?” using a 5-point Likert scale ranging from 1 (very easy to understand) to 5 (not understandable at all).

To evaluate participants’ opinions on the website interface and technical usability, we inquired, “How do you like the design of the website?” and “How well were you able to navigate through the website?” using a 5-point Likert scale ranging from 1 (very good) to 5 (not good at all).

Finally, participants were given the opportunity to provide open-ended feedback by asking, “Do you have any suggestions on how we can improve AinoAid™?” at the end of the survey. Statistical analysis was performed by calculating means (M) and standard deviations (SD), distribution in percent, and correlations for all observed variables in Study 2, to investigate whether age or gender were related to response patterns. The final open-ended response was analyzed using thematic analysis [55], as in Study 1.

### Results Study 2

Table 2 provides the means, standard deviations, and correlations for all observed variables in Study 2. All age groups, ranging from 18 to over 71 years, are represented in the sample, with the majority of respondents falling within their twenties. The means for the measured usability variables indicate a generally positive evaluation across the various aspects, with chatbot safety receiving the highest rating (*M* = 1.07), while helpfulness of interaction and chatbot empathy were rated the lowest (*M* = 2.19). In terms of standard deviations, variability was within an acceptable range (*SD* = 0.26 for chatbot safety, *SD* = 1.07 for helpfulness of chatbot interaction), reflecting an overall positive evaluation of the chatbot by the respondents.

**Table 2:** Mean scores, standard deviation, Pearson correlation coefficient for intercorrelation of the variables in Study 2

	M	SD	1	2	3	4	5	6	7	8	9	10	11
1. Age group	2.91	1.54											
*Chatbot usability*													
2. Helpfulness	2.17	1.07	0,067										
3. Informativity	2.07	1.03	0,011	,744**									
4. Safety	1.07	0.26	0,030	,371**	,341**								
*Chatbot Language*													
5. Simplicity	1.85	0.96	−0,052	,386**	,378**	,232**							
6. Friendliness	1.66	0.82	0,007	,417**	,432**	,230**	,571**						
7. Warmth	2.13	0.99	-,112**	,387**	,435**	,191**	,398**	,601**					
8. Empathy	2.19	1.04	-,159**	,435**	,445**	,292**	,457**	,560**	,764**				
9. Formality	2.13	0.96	−0,068	,427**	,439**	,227**	,500**	,515**	,517**	,569**			
10. Comprehensiveness	1.75	0.88	0,003	,557**	,523**	,378**	,603**	,529**	,409**	,480**	,531**		
*Website usability*													
11. Design	2.00	0.94	−0,057	,468**	,443**	,272**	,400**	,416**	,390**	,413**	,406**	,438**	
12. Navigation	1.81	0.90	−0,028	,365**	,387**	,283**	,444**	,435**	,340**	,393**	,386**	,513**	,518**

*N* = 669. Age group: 1 = 18–30 years, 2 = 31–40 years, 3 = 41–50 years, 4 = 51–60 years, 5 = 61–70 years, 6 = 71 years or older. Variables 3 to 1: low values = positive pole, high values = negative pole

* *p* <.05, ** *p* <.0.01, two-tailed

All correlations between design and usability measures pointed in the same direction, suggesting that they measure related factors, which are generally positively interrelated to a significant degree. Participants’ gender and age group had no noteworthy impact on chatbot evaluation.

A key focus of the analyses in Study 2 was on user suggestions for improvement, aiming to further optimize AinoAid™ as a user-centered support tool for addressing DV. In response to the open-ended question, users demonstrated heightened sensitivity to emotional and linguistic aspects of the chatbot. From their perspective, improvements should primarily focus on enhancing empathy in the chatbot’s tone, adopting a more personal communication style, and creating a more dynamic conversational flow. Specific suggestions included incorporating more frequent follow-up questions, offering encouragement, and using emojis. Additionally, they recommended simplifying the language, significantly shortening responses, and emphasizing key points. These adjustments were seen as essential for making the chatbot more trustworthy, engaging, and comprehensible.

Another concern raised by users is that the chatbot does not always fully understand their input. To uphold a smoother dialogue, it is considered essential that previously shared information be acknowledged and incorporated into the conversation. Content-wise, there is a strong demand for more personalized information and concrete guidance on actionable steps. Many participants stress the importance of receiving targeted referrals to support services rather than general information. Some suggest integrating direct links to external organizations or even enabling direct connections with them. Additionally, some users express a desire for deeper engagement—not just receiving information, but also having the option to communicate directly with support services, emergency responders, or even other individuals affected by DV. Furthermore, a commonly mentioned improvement is the need for broader promotion of the tool across various channels so that the target audience is aware of its existence. Finally, relatively few comments addressed minor improvements regarding the design and usability of AinoAid™.

## Discussion

New digital technologies, such as the AinoAid™ chatbot, have immense potential to improve the support and protection of survivors of domestic violence [36]. Technology can expand the reach of interventions by increasing awareness and providing knowledge, thus encouraging survivors to seek support from appropriate authorities and services [29–31].

We evaluated the potential and usability of the AinoAid™ chatbot through two studies. In the first study, interviewees from different countries shared their views on the chatbot’s potential and desired features, while in the second study, a group of potential users and professionals assessed the usability of AinoAid™ and provided ideas for its further development. Most interviewees viewed the chatbot positively as a potentially significant source of information and support for survivors of DV, but also raised concerns. The second study involved a group of 669 females who tested AinoAid™ and evaluated its usability and language quality. Overall, the respondents rated the usability of AinoAid™ positively, while also asking for simplified language, detailed guidance and enabling of the chatbot to retain information shared earlier in the conversation. They also suggested incorporating the ability to contact various authorities, support services, and emergency responders directly through the chatbot, which, however, would undermine user anonymity.

AinoAid™ offers a rich content, including safety guidance, information on various forms of violence, advice on seeking support, and details about legal procedures, shelters, and other social services. By offering these resources, it serves as a crucial first point of contact, helping survivors of DV become better informed about their situation. Providing information about available services and what to expect from them is a vital first step for survivors of DV to assert their rights [26]. Moreover, AinoAid™ is designed to address some of the barriers that may prevent survivors of DV from seeking help from various service providers. These barriers often intertwine, creating unique constellations in each individual case. For example, individual psychological factors are often intertwined with so-called situational barriers. Among these situational barriers are the cultural context and social relationships in which a survivor’s life is embedded. A survivor of violence, for instance, may experience social isolation when persons in her social network are inclined to accept norms that tolerate violence and the subordination of women [17]. Survivors living in such cultural and social contexts may find AinoAid™ reinforcing their perceptions of their rights and the reprehensibility of violence. However, reporting violence to authorities or relatives, for instance, may worsen survivors’ social isolation if friends and relatives disapprove their actions [46]. In such situations, AinoAid™ can give, nonetheless, advice on self-care, networking and encourage the survivor.

AinoAid™ can help reduce survivors possible lack of awareness of their situation and the lack of knowledge about the availability of various services. Among the most severe barriers are factors that cause survivors to doubt their experiences, leading to self-blame, shame, and low self-esteem [12, 13]. In such circumstances, a conversation with AinoAid™ can help alleviate stress and fear, while improving the survivor’s ability to seek support from various services.

There are structural factors related to the system’s capacity to provide various types of support services and measures that contribute to the survivor’s welfare, health, and security, e.g., geographical distance to services can act as a tangible barrier. Similarly, the lack of services for specific needs, such as support for women with children or the unavailability of interpreters, may limit the survivor’s access to certain services. Negative experiences with particular service providers may, moreover, lead to distrust and reluctance to seek further support [23]. In fact, AinoAid™ assists survivors in establishing realistic expectations regarding the social, medical, and judicial services available to them. However, AinoAid™ cannot address situations where services are simply unavailable in certain areas or for specific groups. There are, nonetheless, also survivors’ misconceptions about the quality and availability of services. These misconceptions can be corrected through the information provided by the AinoAid™ chatbot. Furthermore, even if a survivor has had negative experiences with a particular service provider, AinoAid™ can encourage the survivor to re-contact authorities, regardless of previous negative experiences.

Accordingly, the key factor in the success of the AinoAid™ chatbot is whether the service system can accommodate the potential increase in demand for services. While AinoAid™ can stimulate greater demand for DV-related services, including social services, healthcare, law enforcement, and third-sector support, if these services are unavailable or if their quality does not meet users’ expectations, AinoAid™ may ultimately be perceived as disappointing.

Naturally, the results of this research are subject to further limitations. As the AinoAid™ service is primarily targeted at survivors of domestic violence, they are only presented partly within the sample of Study 2. However, the development of AinoAid™ is an iterative process, meaning that an initial version of AinoAid™ would still be in need of user feedback to improve it towards being appropriate for survivors. To not burden this vulnerable group with a potentially premature service, this group should for ethical reasons be included only at the end of a process for fine-tuning the service, when the chatbot is best trained and all functions are working properly. Instead, general aspects such as usability and most aspects of chatbot language can be equally evaluated by persons not affected by domestic violence. AinoAid™ is, moreover, also conceptualized to support survivors’ friends or bystanders and professionals working in the field of domestic violence. As AinoAid™ has received further training since the survey took place and feedback was used to inform further service development, future research should investigate how survivors who use AinoAid™ in times of need evaluate the service and whether the service has the power to motivate them to approach services of the help system, as intended. In addition, future research should examine the perspectives of DV frontline professionals (e.g., advocates, case managers, and counsellors) on AinoAid™. Such insights would help evaluate the suitability and accuracy of the tool’s content and presentation for DV survivors and clarify whether the service sets realistic expectations of the support system.

The use of technological tools, such as an artificial intelligence chatbot, to improve survivors’ access to support services cannot be implemented without addressing the ethical and security concerns that survivors may have when utilizing such AI-based services. An unconditional prerequisite for the use of the chatbot is its anonymity [32]. Technology must ensure that survivors’ privacy, data security, and safety are not compromised [39, 47]. A second important factor is the immediate and constant availability of the chatbot. This feature is crucial for survivors who are controlled by the perpetrator and may only be able to contact the chatbot occasionally [34]. Besides, a key strength of AinoAid™ is its non-judgmental, impartial nature as the content of the chatbot is curated to not being manipulative, moralizing or accusatory [40]. AinoAid™ is built upon both professional expertise and the experiences and insights of survivors. It provides information and support that are both accurate and relevant for various types of DV survivors. Each survivor is unique and follows her own pace and direction when seeking help. Accordingly, AinoAid™ offers diverse and comprehensive content to meet the needs of a wide range of users, helping them access information and support [39]. A final advantage of an AI-based chatbot is its ability to raise awareness not only among survivors but also to challenge normative and cultural discourses that may attempt to legitimize DV, oppress women, or even blame them.

## Conclusions

This research contributes to the growing body of research exploring the use of technology as a tool for the empowerment and support of women who are survivors of domestic violence. Developed within the framework of the EU project IMPROVE, the AinoAid™ AI-based chatbot demonstrates potential as a valuable source of information and assistance for DV survivors. Our evaluation confirms that AinoAid™ functions as a safe, accessible, and effective gateway to protection and support for survivors of DV. However, ensuring user safety and anonymity, particularly in the context of digital technologies, remains critical for enabling individuals to seek advice and support through AI-based chatbots. The key finding of Study 1, which showed survivors’ openness to the concept of a support chatbot and their positive view on its potential, highlights the value of such tools in complementing existing support services for DV survivors. This is further reflected in the positive evaluations of AinoAid™ in Study 2.

One of AinoAid™’s greatest strengths is its impartiality, which stems from its non-human nature. While this impartiality is highly valued, it also presents a significant challenge in meeting the high expectations for human-like, empathetic, and dynamic conversations in such a sensitive context. Nevertheless, we anticipate that sustained usage and continuous training will enhance the AI’s ability to fully meet the evolving needs of DV survivors.

Despite the valuable contributions of these studies, further research is needed to fully understand the impact of AinoAid™ in addressing and preventing DV. Specifically, examining the perspectives of professionals who interact with the chatbot could offer critical insights into its effectiveness and practical implementation. By incorporating feedback from all potential user groups, AinoAid™ can be further refined to serve as a more comprehensive and effective tool for both, immediate crisis support and long-term intervention.

## Supplementary Information

Supplementary Material 1

Supplementary Material 2

Supplementary Material 3

## Data Availability

The interview guide is available at: https://doi.org/10.5281/zenodo.17670254. The interview data analyzed during the current study are not publicly available, as the informed consent of the interviewees did not include the publication of the interview transcripts because of the sensitive nature of the interviews. They are available from the corresponding author upon reasonable request. The survey data used during the current study are available from the corresponding author upon request. The survey is available at: https://doi.org/10.5281/zenodo.15481033.

## Abbreviations

AI: Artificial Intelligence
API: Application Programming Interface
CMS: Content Management System
CoE: Council of Europe
DPIA: Data Protection Impact Assessment
DV: Domestic Violence
EIGE: European Institute for Gender Equality
EU: European Union
FAQs: Frequently Asked Questions
FRA: European Agency for Fundamental Rights
GDPR: General Data Protection Regulation
GPT: Generative Pre-training Transformer
GREVIO: Group of Experts on Action against Violence against Women and Domestic Violence
IMPROVE: Improving Access to Services for Victims of Domestic Violence by Accelerating Change in Frontline Responder Organisations
IP: Internet Protocol
IPR: Internal Processing Rules
ISO/IEC: International Organization for Standardization/International Electrotechnical Commission
M: Mean
MS: Microsoft
N: Number of people in the sample
NLP: Natural Language Processing
p: Probability
PII: Personal Identifiable Information
RAG: Retrieval Augmented Generation
SaaS: Software as a Service
SD: Standard Deviation
SQL: Structured Query Language
UI: User Interface
VAWG: Violence against women and girls

## Footnotes

1 10.5281/zenodo.15481033.
