# Supplementary figures and images for: From needs assessment to usability testing: evaluating the AinoAid™ chatbot for domestic violence support
Source: BMC Womens Health. 2025 Dec 12;26:31. doi: 10.1186/s12905-025-04202-3 (PMC12817862; doi:10.1186/s12905-025-04202-3)

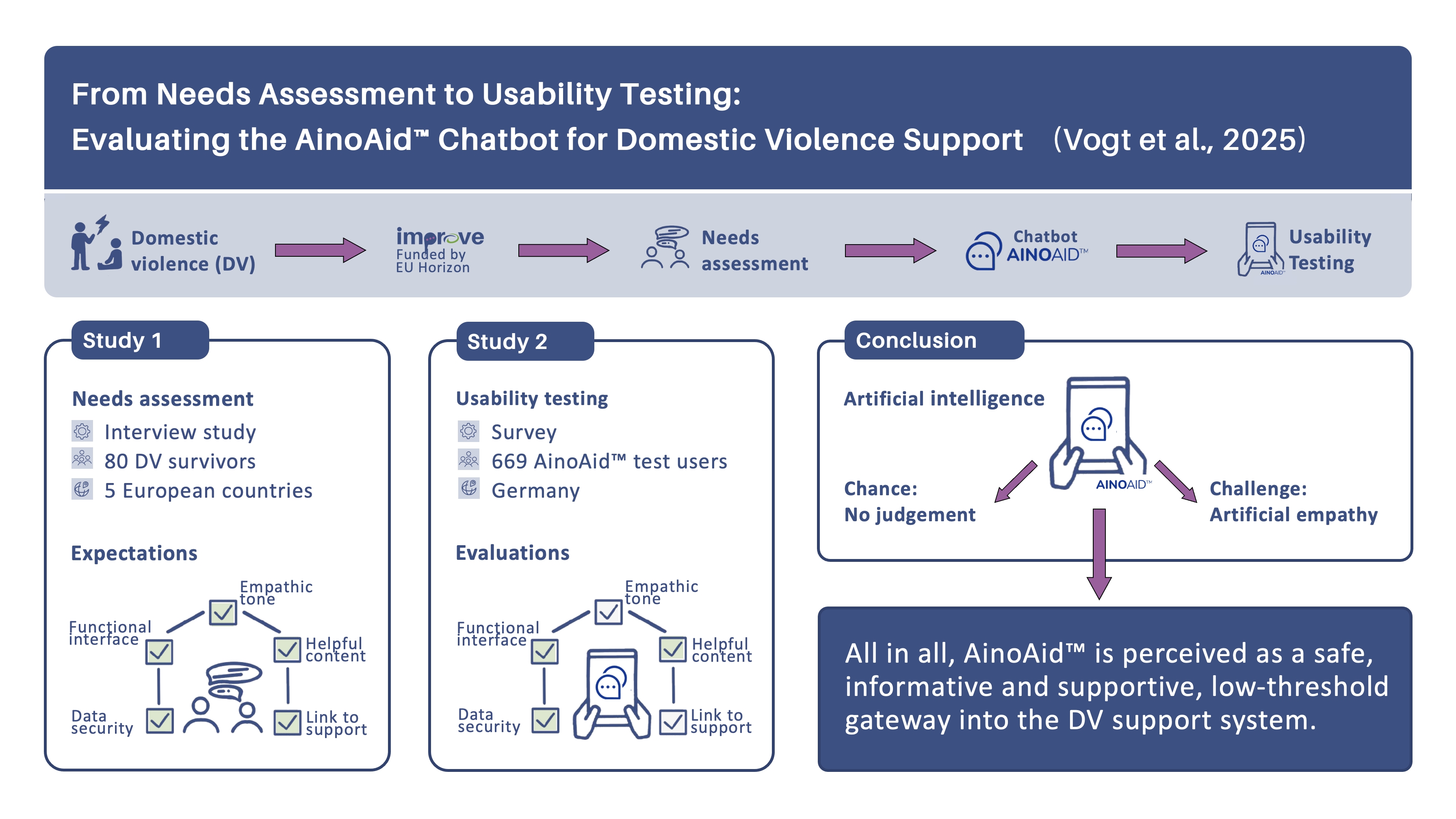

Supplement: Supplementary file 1 — Supplementary Material 1 [file 12905_2025_4202_MOESM1_ESM.jpg]
