# Supplementary material for: From needs assessment to usability testing: evaluating the AinoAid™ chatbot for domestic violence support
Source: BMC Womens Health. 2025 Dec 12;26:31. doi: 10.1186/s12905-025-04202-3 (PMC12817862; doi:10.1186/s12905-025-04202-3)
Supplement: Supplementary file 2 — Supplementary Material 2 [file 12905_2025_4202_MOESM2_ESM.pdf]

Authors: Catharina Vogt, Natalie Köpsel, Stefanie Giljohann
Date: 21.05.2025

Survey on user experience of AinoAid™ chatbot

Personal Data

Please indicate the gender you identify with:

- ◯ Female
- ◯ Male
- ◯ Diverse

Please enter your age group:

- ◯ <18 years
- ◯ 18-25 years
- ◯ 26-30 years
- ◯ 31-40 years
- ◯ 41-50 years
- ◯ 51-60 years
- ◯ 61-70 years
- ◯ >71 years

Questions about the AinoAid™ chatbot and the website

Which of the following statements applies?

- ◯ You can only chat in German with AinoAid™.
- ◯ With AinoAid™ you can also chat in other languages.

What do you confirm when you open the AinoAid™ chat?

- ○ That AinoAid™ saves chat histories/conversations.
- ○ That I have to delete all cookies after chatting.

General questions about the AinoAid™ chatbot

How helpful did you find the exchange with the chatbot?

Very helpful	○	○	○	○	○	Not helpful at all

How informative did you find the chatbot's answers?

Very informative	○	○	○	○	○	Not informative at all

How did the chatbot's language style work for you?

Simple	○	○	○	○	○	Complicated
Friendly	○	○	○	○	○	Unfriendly
Warm	○	○	○	○	○	Cold
Sensitive	○	○	○	○	○	Distanced
Pleasantly formal	○	○	○	○	○	Too formal

How comprehensible did you find the chatbot's answers?

Very comprehensible	○	○	○	○	○	Not comprehensible at all

Did you feel it was safe to use the chatbot?

- ○ Yes
- ○ No

General questions about the AinoAid™ website

How do you like the design of the website?

Very good ◯ ◯ ◯ ◯ ◯ Not good at all

How well were you able to navigate through the website?

Very good ◯ ◯ ◯ ◯ ◯ Not good at all

Background experience

In the following, we would like to ask you for some information about your experience with domestic violence.

Did you have anything to do with domestic violence before this study? (multiple answers are possible)

- ☐ I was/am affected by domestic violence myself.
- ☐ Relatives/acquaintances of mine were/are affected by domestic violence.
- ☐ I am involved with the topic of domestic violence through my work.
- ☐ No, I had no contact with the topic of “domestic violence” before this study.
- ☐ None of the suggested answer options apply.
- ☐ No answer.

Have you already dealt with the topic of domestic violence in depth?

- ◯ Yes
- ◯ No
- ◯ No answer.

If so, how did you become involved with the issue of domestic violence?

Voluntary information.

Have you ever used another chatbot to help with domestic violence?

- ◯ Yes
- ◯ No
- ◯ No answer.

What was the name of this chatbot?

Voluntary information.

What advantages and disadvantages did this chatbot have in comparison with AinoAid™?

Voluntary information.

Do you have any comments on the survey or suggestions on how we can improve AinoAid™?

Voluntary information.

This research was conducted as part of the IMPROVE research project, funded by the European Commission's Horizon Innovation Action funding scheme under Grant Agreement No. 101074010.
