# Supplementary material for: From needs assessment to usability testing: evaluating the AinoAid™ chatbot for domestic violence support
Source: BMC Womens Health. 2025 Dec 12;26:31. doi: 10.1186/s12905-025-04202-3 (PMC12817862; doi:10.1186/s12905-025-04202-3)
Supplement: Supplementary file 3 — Supplementary Material 3 [file 12905_2025_4202_MOESM3_ESM.pdf]

Code: In/______/_________
Initials/Interview Nr.

IMPROVE-Project

Semi-structured interview guide

How to use the semi-structured interview guide:
The present guidelines serve as a baseline version for the national translations. The majority of the content can and should be adapted to the linguistic and cultural peculiarities as well as to the anticipated interview setting of the respective country. Consequently, the introduction and sub-questions can be rephrased as needed. The wording contained in this document serves only as a rough template. When translating, however, care should be taken to maintain the thematic orientation. The goal descriptions given for each section serve as orientation.
In contrast, the main questions in bold should be translated as accurately as possible and not be skipped, omitted or reworded. They represent the core content that spans the entire research project.

Before the interview:
1. Introduction of the project and thanks for participation
2. Explain confidentiality and privacy.
3. Fill in consent form
4. Explain benefits of audio recording: Enhances the interview process, more accurate description and reproduction of expertise than just notes etc
5. Talk about Participants' rights regarding ownership of data and data protection.
6. Overview of the interview (Interview Introduction)
    6.1. How long will it approximately take.
    6.2. Which topics will be discussed and roughly in which order.
    6.3. Emphasise that there are no right or wrong answers and that participants are welcome to “deviate” in their narratives and talk about everything they feel that is important in relation to the topic.
    6.4. Emphasise that participants do not need to answer any question the do not want to or talk about topics they feel uncomfortable with.
    6.5. Emphasise that participants can take a break or stop the interview.

6.6. Ask if participants have any questions before the start, if not start with opening question

6.7. If you feel its necessary to “lighten the atmosphere” instead of starting with the first question, it is also possible to start the conversation by asking the participants to introduce themselves. However, this may alter the suggested sequential order as participants may already introduce relevant topics. In order to honour this relevance framework set by the participants, it is necessary to pick up on these topics and discuss them in more detail, and to consider other questions only in the further course of the interview.

Introduction

Thank you for participating in our study on iimproving access to services for victims of domestic violence. Since you have agreed to the audio recording, I will start the taping now. Please note, that I will also take notes during the interview.

To begin with, I would like to talk about the process from the moment you decided to seek help to the moment you first came into contact with a help organisation, such as a women's shelter or a violence protection centre. Here I am particularly interested in when the relationship reached the stage where you decided to seek help, and whether you found it difficult or easy to find out where to turn to get the help that suited your needs. In the second part of the interview, I would like to talk in detail about your experiences in contact with the support services, i.e. whether you felt appreciated, whether the support met your expectations and how seeking support has impacted your life. Following on from this, I would be interested to hear if you have any suggestions on how to better support people who face a similar situation to yours in the future. In particular, I would like to talk to you about what needs to be done so that people can get in contact with support organisations or intervention centres more quickly and safely. At the end of the interview, I would like to hear your opinion about the chatbot we are developing in the research project. A chatbot is a application that can be accessed through different devices, such a mobile phones and laptops. It can talk to people and provide them with help and information automatically. We are interested in whether you think that such a chat-bot could be useful as an additional first point of contact for people affected by violence, and

whether and under what conditions you would use such a chat-bot.

Before we begin, I would like to emphasise that since the interview is about your experience, you are to decide the direction and content of the interview. I would therefore ask you to talk about all aspects of the topic that you think are important even if those may not seem to be directly related to my questions.
If you do not have any questions at this stage, I would start with my first question.

Please note: This semi-structures guide is a part of a larger interview guide used for data collection in T1.2 of the IMPROVE Project. It only includes the questions on AI.

<table>
<tr><td colspan="2">Section 4. Chatbot solution for providing victim support</td></tr>
<tr><td>Aim:
Appropriateness of Chatbot tool, expectations of victims towards such a tool, as well as factors influencing its use</td><td>
1. As already mentioned, one goal of the IMPROVE project is to develop a chatbot AINO that will help victim-survivors seek support by lowering barriers to entry and providing relevant information. AINO provides an easy, reliable and trustworthy source for finding, accessing and using DV-related information. The user gets a human-type of conversation for psycho-social support and guidance through the chat and easy-to-find information for more and relevant contact details from the knowledge base. So a chatbot is a text-based dialogue system that uses artificial intelligence, and I would like to know whether you would use such a chatbot. What should such a chatbot have to be able to do, and how it would have to be designed in order for you to use it as a platform that helps victims of violence get further support. First of all, however, I would like to know if you have ever used a chatbot, and if so, what for?

2. So, in general how do you feel about a chatbot helping victim survivors understand what they are undergoing and how to get help?

o What do you think could be the advantageous of a chatbot?
o What do you think might discourage victims from using a chatbot?
o What would be your personal major concerns about using a chatbot?
o In any case, what must such a chatbot be able to do so that victims would use it?
o Are there any specific questions or content (e.g. information on what violence actually is) that such a chatbot should provide to users?
o What do you think would be a good initial question for the chatbot when you first launch it?
o Do you think such a chatbot could also be useful beyond the initial counselling and information provision in the further course of the support and healing process?
</td></tr>
</table>

	**3. Would you be interested in helping along the development of this Chatbot further (providing feedback at certain stages throughout the development process)?**<br><br>**4. I would now like to ask a few questions about the design and functions of a chatbot?**<br><br>o We already talked about trust-building an important aspect in victim support. What would make you trust the chatbot?<br><br>o If you wanted to use such a chatbot, would you rather talk and listen to the bot or write with the bot?<br><br>o What kind of tone (e.g., male, female) of voice should the chatbot have in order to make it professional and trustworthy and sound friendly?<br><br>o Do you think there should be a possibility to be connected directly from the chatbot to the police/hospital/women's shelter or violence protection centre/send a message directly to these organisations?<br><br>o Should it be possible to download the conversation with the chatbot or send it as a text message/email, e.g. to your own email address or friends etc.?<br><br>o Would you prefer the chatbot to be available on websites, e.g. information websites of authorities or violence protection centres or through channels, such as WhatsApp, Telegram, Facebook messenger, or would you prefer it to be available as an application for mobile phones etc?<br><br>o Do you think the chatbot should give advice and information on how victims can behave in emergency situations and what they should do, e.g. if someone is very scared due to the threat of severe violence?<br><br>o If the chatbot were a mobile phone application, do you think it should function like a kind of emergency button?<br><br>o Finally, do you have any ideas on how to promote such a chatbot so that as many people as possible would become aware of it?

Closure

Is there anything else you would like to add, something that has not been addressed, that I have not asked, but that would be important?

Thank you for your time and participation
